# rs-fMRI and machine learning for ASD diagnosis: a systematic review and meta-analysis

**DOI:** 10.1038/s41598-022-09821-6

**Published:** 2022-04-11

**Authors:** Caio Pinheiro Santana, Emerson Assis de Carvalho, Igor Duarte Rodrigues, Guilherme Sousa Bastos, Adler Diniz de Souza, Lucelmo Lacerda de Brito

**Affiliations:** 1 Institute of Systems Engineering and Information Technology, Federal University of Itajubá (UNIFEI), Itajubá, 37500-903 Brazil; 2 Department of Computing, Federal Institute of Education, Science and Technology of South of Minas Gerais (IFSULDEMINAS), Machado, 37750-000 Brazil; 3 Institute of Mathematics and Computation, Federal University of Itajubá (UNIFEI), Itajubá, 37500-903 Brazil; 4 Luna ABA, São José dos Campos, 12246-000 Brazil

**Keywords:** Diagnostic markers, Biomarkers, Diseases, Neurology, Mathematics and computing

## Abstract

Autism Spectrum Disorder (ASD) diagnosis is still based on behavioral criteria through a lengthy and time-consuming process. Much effort is being made to identify brain imaging biomarkers and develop tools that could facilitate its diagnosis. In particular, using Machine Learning classifiers based on resting-state fMRI (rs-fMRI) data is promising, but there is an ongoing need for further research on their accuracy and reliability. Therefore, we conducted a systematic review and meta-analysis to summarize the available evidence in the literature so far. A bivariate random-effects meta-analytic model was implemented to investigate the sensitivity and specificity across the 55 studies that offered sufficient information for quantitative analysis. Our results indicated overall summary sensitivity and specificity estimates of 73.8% and 74.8%, respectively. SVM stood out as the most used classifier, presenting summary estimates above 76%. Studies with bigger samples tended to obtain worse accuracies, except in the subgroup analysis for ANN classifiers. The use of other brain imaging or phenotypic data to complement rs-fMRI information seems promising, achieving higher sensitivities when compared to rs-fMRI data alone (84.7% versus 72.8%). Finally, our analysis showed AUC values between acceptable and excellent. Still, given the many limitations indicated in our study, further well-designed studies are warranted to extend the potential use of those classification algorithms to clinical settings.

## Introduction

Autism Spectrum Disorder (ASD) is a life-long neurodevelopmental condition associated with the atypical development of the brain. Individuals in this group, in general, present a slow development in certain activities when compared to individuals of Typical Development (TD)—such as speech, motor coordination, and social activities—and difficulties communicating and relating to others^1,2^.

Despite being considered a neurological disorder, the diagnosis of ASD remains exclusively based on behavioral criteria^3^. This may be due to the great heterogeneity within the population, possibly reflecting an enormous amount of different neurodevelopmental etiologies^4,5^.

Typically identified in early childhood, ASD’s development is believed to have genetic and environmental roots^6,7^. According to recent publications^8,9^, the former accounts for approximately 80% of the cases. Also, epidemiological studies suggest an increase in its global prevalence in recent years, and a systematic review published in 2012 estimated it to be about 0.62%^10^.

The impact of this condition on the quality of life extends beyond the affected individual to the entire family. For example, parents of children with ASD report higher stress levels than parents of children with other disabilities^11^. Also, the majority of researches regarding autism is based on data from high-income countries. This creates inequities across the world in access to services and supports^12^.

On the other hand, given the brain’s plasticity during the first years of life, early detection paired with early treatment would have considerably stronger benefits than later treatments^13,14^.

The gold standard diagnosis of ASD is based on a differential diagnostic examination by an experienced clinician, frequently supported by tools such as the Autism Diagnostic Interview-Revised (ADI-R)^15^—a standardized caregiver interview—and the Autism Diagnostic Observation Schedule (ADOS/-2)^16^—a semi-structured standardized observation for individuals with suspected ASD. Therefore, this is a long and time-consuming process that requires a multi-disciplinary team to assess information from various sources^17,18^.

In recent years, Machine Learning (ML) classifiers have been increasingly applied to neuroimaging data to diagnose psychiatric disorders, including ASD. Those classification methods hold the promise of facilitating and speeding up the diagnostic process^19,20^.

Throughout the different types of neuroimaging data, the resting-state functional Magnetic Resonance Imaging (rs-fMRI) is increasingly used to investigate neural connectivity and identify biomarkers of psychiatric disorders. It is based on spontaneous fluctuations in the Blood Oxygenation Level-Dependent (BOLD) signal obtained through a non-invasive and relatively fast acquisition process. Also, the rs-fMRI is task-free—requiring no active and focused participation of the patient—and the data can be easily combined to generate large databases^19,21,22^.

We can highlight the Autism Brain Imaging Data Exchange (ABIDE) as one example of such databases. Together, the first^23^ and second^24^ versions of the repository (ABIDE I and ABIDE II) aggregate rs-fMRI and corresponding structural data of more than 2000 individuals with ASD and of TD collected across more than 24 international brain imaging laboratories.

Studies using rs-fMRI data have revealed brain functional connectivity patterns that could serve as biomarkers for classifying depression^25^, Parkinson’s disease^26^, Attention Deficit Hyperactivity Disorder^27^, ASD^28^, and even age^29^. However, the reproducibility and generalizability of these approaches in research or clinical settings are debatable. There are many potential sources of variation across studies, and its effect on diagnosis and biomarker extraction is still poorly understood^22,30^.

Therefore, we conducted a systematic review and meta-analysis of studies that used ML classifiers based on rs-fMRI data to distinguish patients with ASD from individuals of TD. We aimed to critically review the current literature on this area based on the following research questions:

- Which ML techniques are used to classify ASD and TD individuals based on rs-fMRI?
- What are the results obtained by the studies using these approaches?
- Which methodological differences are associated with the performance measures obtained throughout the publications?
- The approaches are robust enough to be applied in a clinical setting?
- What are the aspects that still need to be investigated?

## Results

We searched for articles using four digital libraries and a backward snowballing approach^31^—i.e., looking for new relevant articles in the references of the selected ones. Three authors conducted a selection process based on specific inclusion and exclusion criteria. Articles were pre-selected if at least one author concluded they should be, and the final selected papers were defined by consensus.

Data extraction was performed on the selected articles using a standardized data extraction sheet, considering only one result per independent sample. Publications with enough information to obtain measures of True Positive (TP), True Negative (TN), False Positive (FP), and False Negative (FN) were included in the meta-analysis. Also, the Quality Assessment of Diagnostic Accuracy Studies (QUADAS-2)^32^ was applied to assess studies’ methodological quality. More details of our methodology can be found in “Methods” section.

The following subsections present the characteristics of the studies selected for (i) the systematic review and (ii) the meta-analysis, and the results of (iii) the quality assessment and (iv) the quantitative meta-analysis.

### General study characteristics

**Figure 1:**
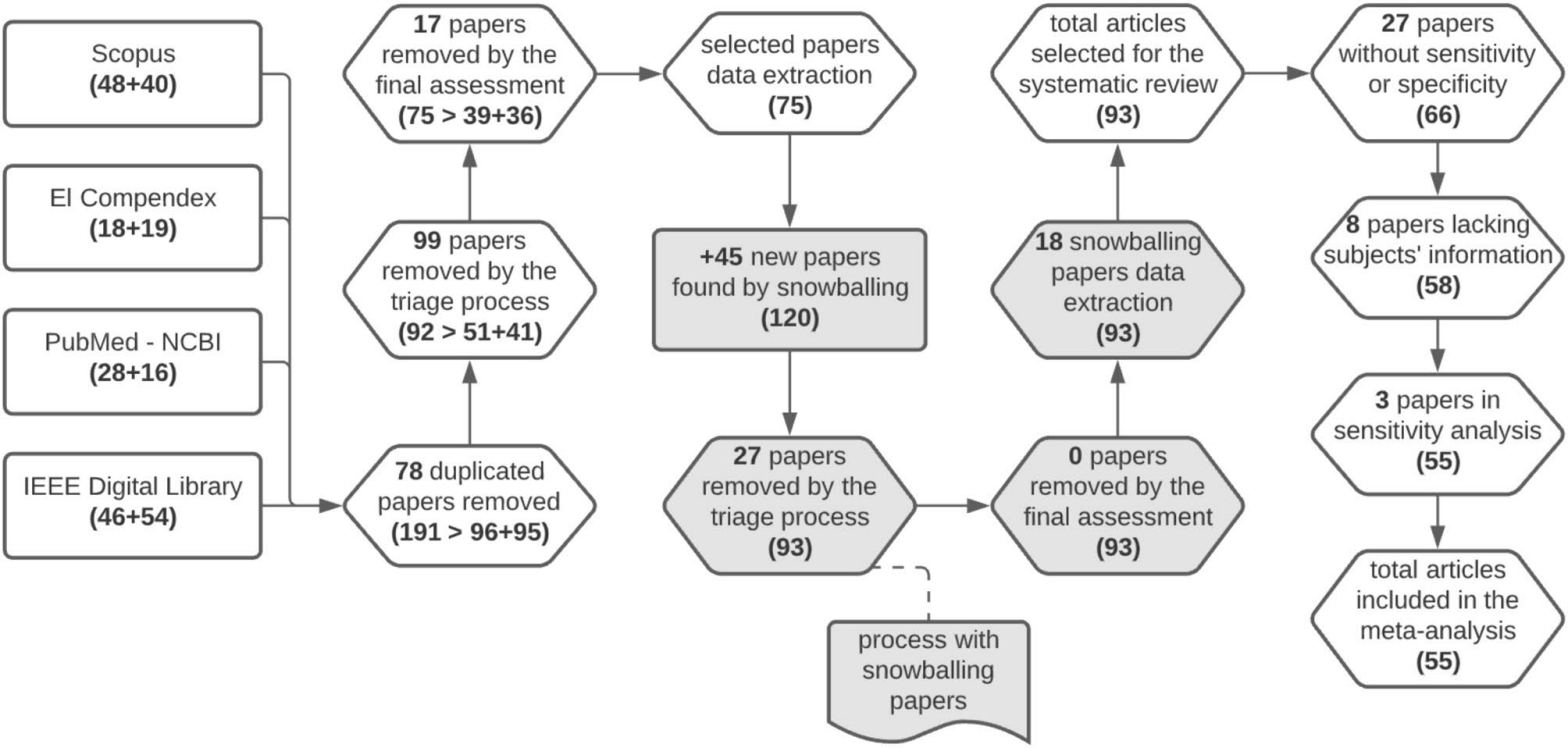
Screening and selection of studies according to inclusion and exclusion criteria at different stages of the meta-analysis. The numbers between parentheses indicate the total of articles remaining after each step. The numbers separated by $+$ indicate the count of articles from the first and second search, respectively. Created with Lucidchart Free https://www.lucidchart.com.

A total of 93^5,19,22,30,33–121^ studies were selected for the systematic review. Figure 1 summarizes our selection methodology and the details according to the screening stage. Also, the final publications identified and selected can be found as Supplementary Tables S1–S3.

**Figure 2:**
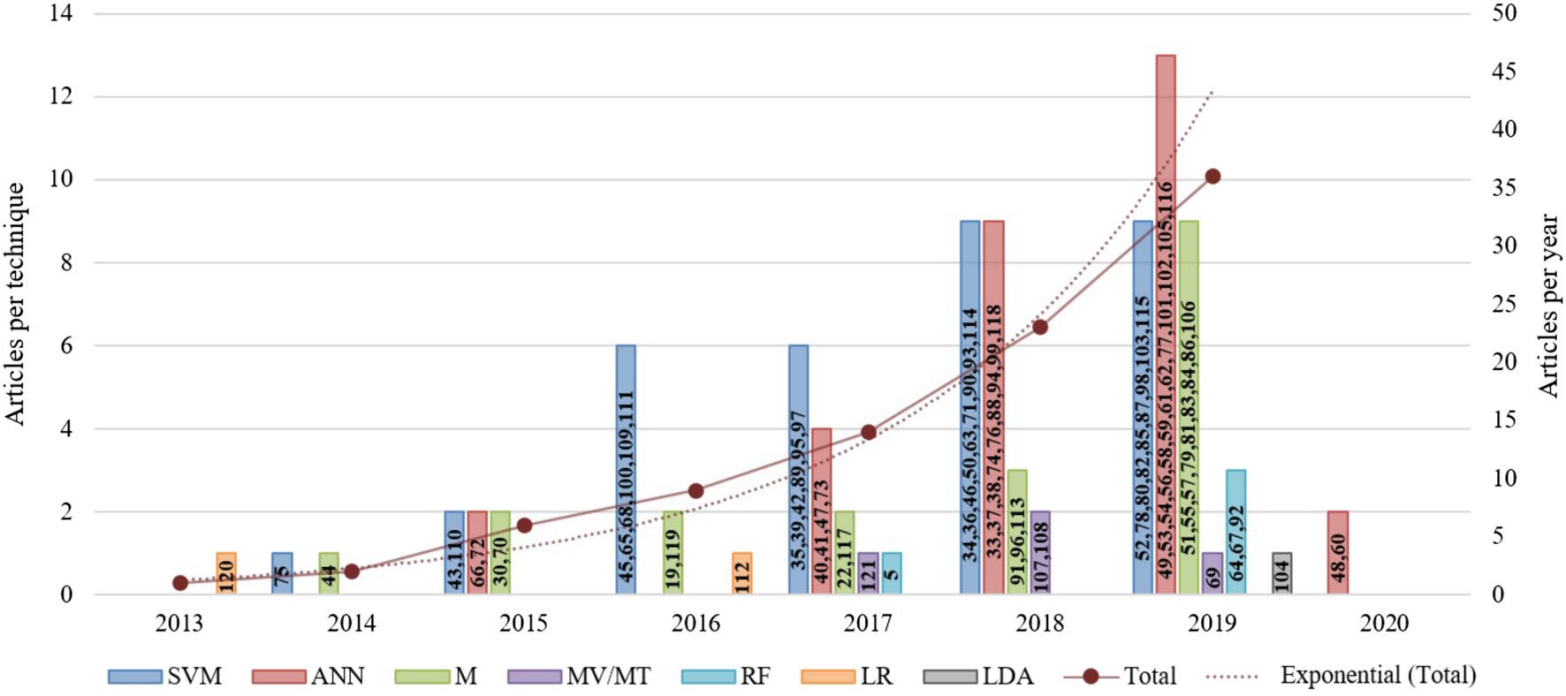
Distribution of the selected studies by year of publication and type of ML technique used (*MV/MT* multiview/multitask learning, *RF* Random Forest, *LR* Logistic Regression; *LDA* Linear Discriminant Analysis). The numbers inside the bars indicate each article. Created with Microsoft Excel 2019.

All the 93 studies were published between 2013 and 2020 and used samples that varied from 24 to 2352 individuals. The most commonly applied ML techniques for classification were Support Vector Machine (SVM, $n = 33$) and Artificial Neural Network (ANN, $n = 30$), followed by studies that used more than one technique (M, $n = 19$). Figure 2 shows the distribution of the selected articles by year and ML technique used, whereas Table 1 shows the general characteristics of the included studies.

**Table 1:** General characteristics of the studies selected in the systematic review (SR) and the studies and samples included in the meta-analysis (MA).

Characteristics	Studies (SR)	Studies (MA)	Samples (MA)
Total	93	55	132
**ML technique**			
SVM	33	27	54
L-SVM	−	14	33
Other	−	13	21
ANN	30	13	44
CNN	−	5	16
Other	−	8	28
M	19	2	2
MV/MT	4	3	15
RF	4	2	2
LR	2	3	4
LDA	1	2	8
Ridge	−	1	1
XGB	−	1	1
Affine	−	1	1
**Dataset**			
ABIDE (any version)	79	45	121
ABIDE without version	34	21	41
ABIDE I − preprocessed	34	19	54
ABIDE I + ABIDE II	7	5	26
ABIDE I	2	0	0
ABIDE II	2	0	0
UMCD	3	2	2
NDAR	3	2	2
Own sample	3	4	4
Own sample + ABIDE	3	2	2
Others	2	1	1
**Type of data**			
Only rs-fMRI	73	49	114
rs-fMRI plus other types of brain imaging data	11	3	14
rs-fMRI plus phenotypic information	9	3	4
**Sex of the subjects**			
Males and females	62	37	80
Not enough information	26	13	44
Only males	5	6	8
**Age of the subjects**			
Both above and below 18 y.o.	42	26	62
Not enough information	28	10	25
Below 18 y.o.	20	20	39
Above 18 y.o.	3	4	6
**FIQ of the subjects**			
Not enough information	−	33	90
Both high- and low-functioning	−	14	30
Only high-functioning	−	8	12

Note that for the dataset, sex, and age of the subjects, the sum of the column Studies (MA) is greater than 55 due to articles with multiple samples included in different categories.

*L-SVM* Linear SVM, *CNN* Convolutional Neural Network, *Ridge* Ridge classifier, *XGB* Extreme Gradient Boosting, *Affine* Affine-Invariant, *y.o.* years old, *FIQ* Full Intelligence Quotient.

Significance values are given in italics.

Almost 85% of the studies ($n = 79$) extracted their samples from versions of the ABIDE, specially ABIDE I preprocessed ($n = 34$) or ABIDE without specifying the version ($n = 34$). The other articles used data from the UCLA Multimodal Connectivity Database^122^ (UMCD, $n = 3$), the National Database of Autism Research (NDAR, $n = 3$) [http://ndar.nih.gov], own samples ($n = 3$), own samples and ABIDE ($n = 3$), others ($n = 2$).

The majority of the studies ($n = 73$) used only rs-fMRI data for classification. Beyond that, some studies used other types of brain imaging data ($n = 11$) or phenotypic data ($n = 9$).

Regarding the subjects’ characteristics, we found studies that included both males and females ($n = 62$), only male subjects ($n = 5$), and studies that did not present enough information regarding the sex of the selected individuals ($n = 26$). Furthermore, there were samples with subjects both above and below 18 years old ($n = 42$), only below 18 ($n = 20$), only above 18 ($n = 3$), and studies without enough information ($n = 28$).

### Studies included in the meta-analysis

From the 93 studies selected for the systematic review, 27^33–59^ did not report any data regarding sensitivity or specificity and were excluded from the meta-analysis. Five articles^60–64^ did not present the exact number of TD and ASD subjects on the sample or the test set, making it impossible to calculate the measures necessary for the meta-analysis. Two articles^65,66^ defined specific sample percentages as training or test sets and performed some random trials. Thus, it was impossible to determine the exact number of subjects in the test set nor the proportion of ASD and TD subjects. In another study^67^, seven subjects had corrupted rs-fMRI imaging files, so they were included in the structural MRI (sMRI) analysis and excluded from fMRI analysis and sMRI-fMRI modalities fusion. However, the study did not inform from which group (ASD or TD) those subjects were.

Two articles^68,69^ presented their results through bar charts without showing the exact sensitivity and specificity values, so we decided not to include them in the meta-analysis. In another publication^5^, an RF was used as the classifier, and sensitivity and specificity measures were presented only for the external validation data-set. The main results from the article were obtained through the out-of-bag (OOB) error—by subsampling with replacement to create training samples for each tree, the excluded data are used for testing, and the mean of the results generates the OOB error—but only the accuracy was reported. Since the results from the validation data-set and the ones obtained using the OOB error presented high variation, we decided not to use the results from this article. However, those three articles were included in a sensitivity analysis to assess their effect on the meta-analysis results.

Finally, 55 studies^19,22,30,70–121^—published between 2013 and 2019—provided sufficient data for a quantitative meta-analysis. Note that the information presented in the rest of this section is related to the main results extracted from those studies, as described in “Data extraction” section.

Since some studies comprise multiple samples, a total of 132 independent samples were extracted, with sensitivity and specificity ranging from 37.5% to 100% and 20% to 100%, respectively. About 85% of the studies extracted their samples from versions of the ABIDE, corresponding to 93% of the samples. A coupled forest plot of sensitivity and specificity for all the samples included in the meta-analysis can be found as Supplementary Fig. S1. Table 1 also presents the general characteristics of the studies and samples included.

From all samples, 123 used ABIDE data in the test sets. To better understand their overlap, we calculated how many samples used each ABIDE site. Sixteen of them did not present enough information to define which sites they came from. ABIDE I was used in 100 samples, while ABIDE II was used in nine. Note that some samples comprised both versions of the database and, for this analysis, we defined the ABIDE version according to the sites’ names presented if the articles did not directly specify which ABIDE they used.

In the case of ABIDE I, each site was used from 13 to 33 times throughout the samples, with a mean of 19.2 samples using each site. Besides, 60 samples used only one site, 16 samples used two sites, and seven samples used all ABIDE I sites. As for ABIDE II, ignoring the sites that were not used, each site was used from one to three times, with a mean of 1.5 samples using each site. In this case, six samples used only one site.

It is worth noticing that a site being used in 33 samples does not mean a complete overlap 33 times since each sample used a different number of subjects from each site. Even knowing the number of subjects used from each site in each sample and the total number of subjects available in each site, one cannot assess the extent of the overlap once it is not known which specific subjects were used. In fact, only two of the articles^22,96^ presented which subjects from ABIDE were used in their studies.

Beyond that, less than 50% of the studies ($n = 24$ articles/61 samples) presented the mean age, almost 24% ($n = 13/44$) had not enough information regarding the sex of the subjects, and only 20% ($n = 11/18$) presented the mean FIQ of their ASD and TD samples. Also, only 1 of the samples^112^ used in the meta-analysis—a multi-site dataset from Japan—was not from North America or Europe.

**Figure 3:**
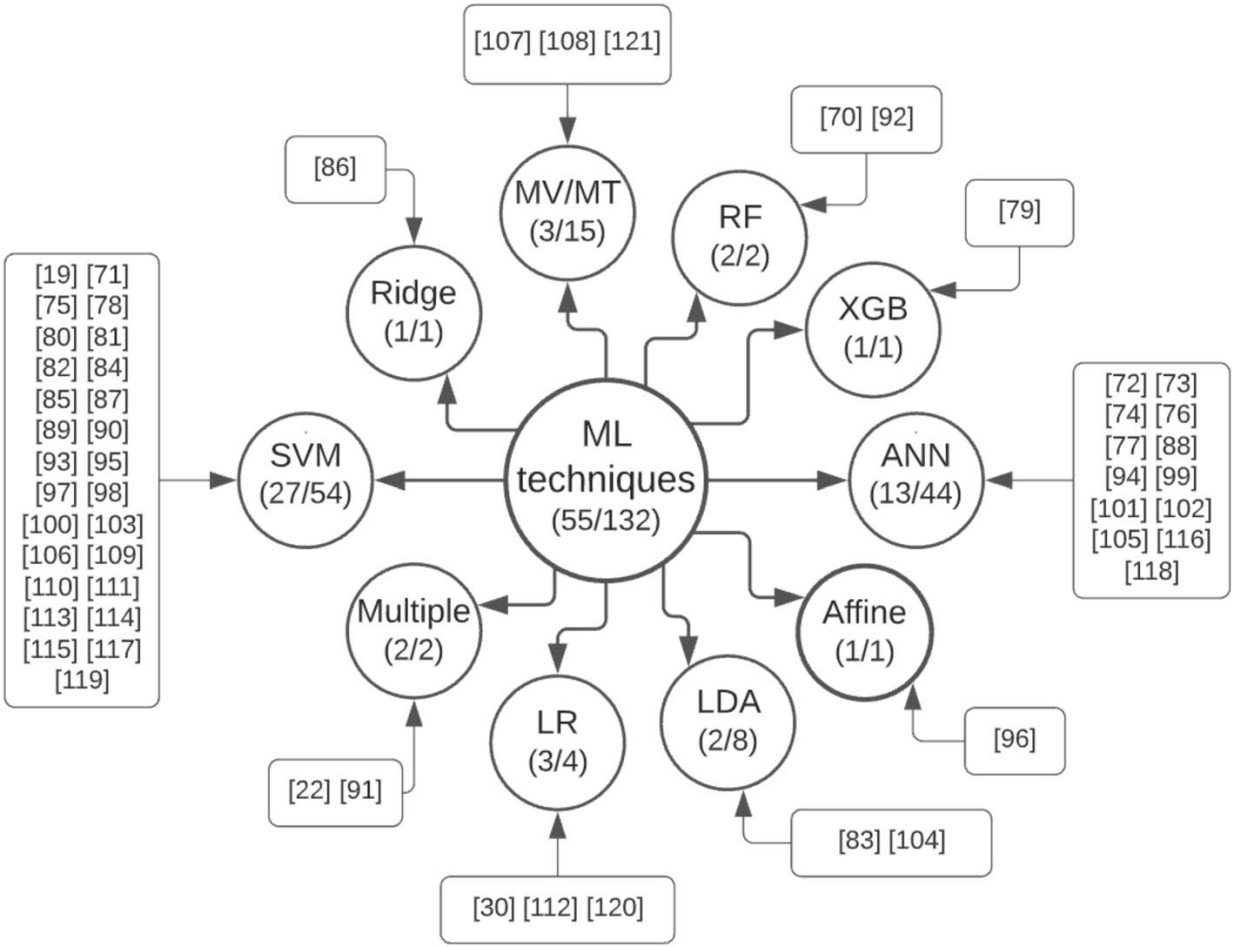
Conceptual map of ML techniques used throughout the articles selected for meta-analysis (number of articles/number of samples). Created with Lucidchart Free https://www.lucidchart.com.

Most of the articles defined Regions of Interest (ROIs) of the brain to reduce the dimensionality of their features using a priori atlases or combinations of them ($n = 41/101$), especially the Automated Anatomical Labelling^123^ in versions of 90 (AAL90, $n = 5/8$) and 116 ROIs (AAL116, $n = 13/28$) and the Craddock atlas with 200 ROIs^124^ (CC200, $n = 6/22$).

Throughout the studies, there were various approaches to extract features from the data, from different points of view, and in varying levels of complexity. However, the most used type of features consisted of estimating functional connectivity (FC) patterns^19^ using the Pearson Correlation (PC) between all pairs of averaged time-series. Those studies used the PC either on its original version ($n = 8/28$) or normalized by Fisher transformation ($n = 7/20$).

According to the main results, the techniques used for classification are presented through a conceptual map in Fig. 3.

### Quality assessment

The QUADAS-2 is a tool designed to assess the quality of primary diagnostic accuracy studies, evaluating if systematic flaws or limitations might distort their results—i.e., if there is a Risk of Bias (RoB)—and if these studies apply to the review’s research question^32^.

**Figure 4:**
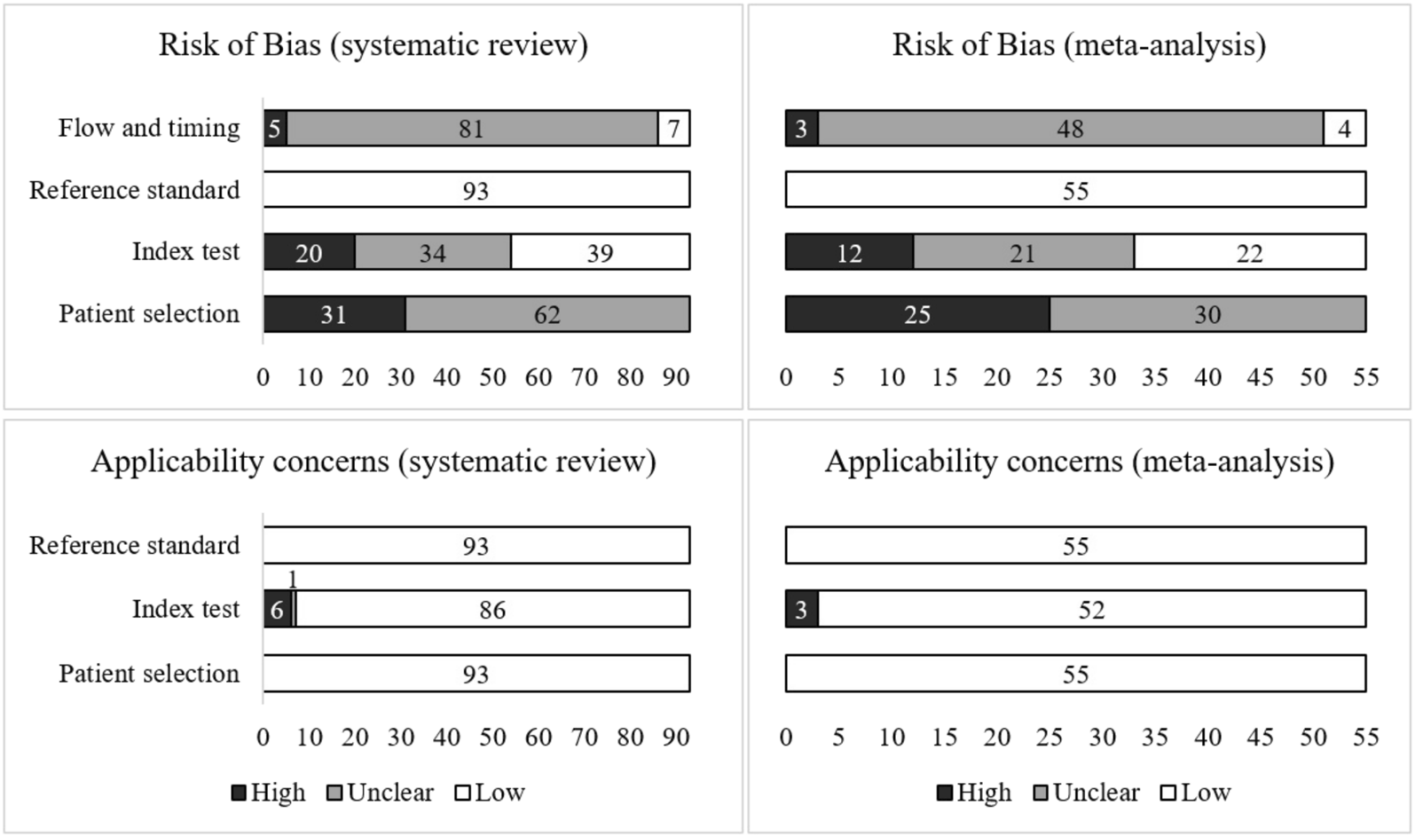
Risk of bias and applicability concerns by domain in QUADAS-2 for all the studies selected for the systematic review (left) and considering only the ones included in the meta-analysis (right). Created with Microsoft Excel 2019.

Figure 4 shows the distribution of the results of QUADAS-2 considering all the publications selected for the systematic review or the studies included in the meta-analysis (see Supplementary Table S4). The only difference in the results between the two applications of the tool was in the RoB by index test. Since some of the results in the articles did not have enough information to be used in the meta-analysis, three studies^70,71,117^ showed low RoB by index test when assessed as a whole, but when considering only the results used for the meta-analysis they presented unclear RoB.

We can highlight that none of the studies was considered to have a low RoB by patient selection domain. Most of them had an unclear RoB (62 out of 93 in the first and 30 out of 55 in the second application) since they used databases such as the ABIDE and did not present details regarding the recruitment of the subjects nor sufficient information of the characteristics of the subjects selected. The remaining articles were shown to have a high RoB due mainly to the selection of subjects in restricted intervals of age and intelligence quotient (IQ) or the exclusion of female subjects.

The great majority of the studies were considered to have an unclear RoB by flow and timing domain (81 and 48 studies) mainly because they did not present the interval between the application of the index test and the reference standard nor sufficient information to conclude if all subjects received the same reference standard.

All of the articles were shown to have a low RoB by reference standard domain given that we considered the reference standards used in databases such as the ABIDE as reliable even if the article did not present exactly what reference standards were used. For the same reason, all of the studies were assessed to have low concerns regarding applicability by the same domain. We highlight that the reference standards used throughout the studies were similar to those used in ABIDE^23^: combining clinical judgment and diagnostic instruments; clinical judgment only; or diagnostic tools only.

More than half of the articles were considered to have an unclear or high RoB (54 and 33) by the index test domain. Also, most of the studies (86 and 52) were assessed to have low concerns regarding applicability by the same domain. Finally, all of the articles were shown to have low concerns regarding applicability by the patient selection domain.

According to the first application of the QUADAS-2 tool, the RoB was judged as high in at least one category in 42 studies, and 12 studies presented a high RoB in at least two domains. Finally, according to the second analysis, the RoB was judged as high in at least one category in 29 studies, and 9 studies presented a high RoB in at least two domains.

### Diagnostic accuracy

As commented before, there is a literature limitation regarding sample overlap. This should be taken in consideration while analyzing the quantitative results presented in this section. The implications and other considerations regarding this issue can be found in “Limitations” section.

**Figure 5:**
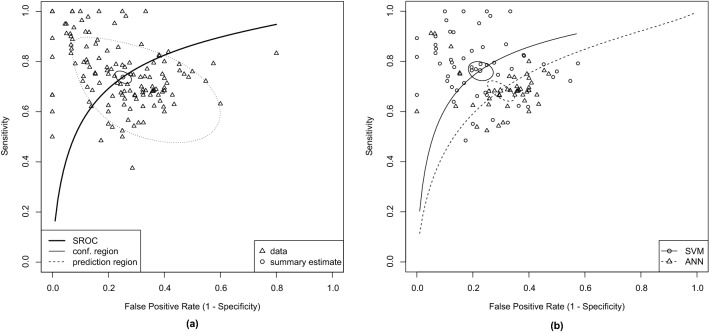
SROC curves of all the included studies with summary estimate **(a)** and the studies using SVM and ANN with their summary estimates and confidence region **(b)**. Created with R Statistics^125^ version 4.1.1 using the package mada^126^ version 0.5.10.

Using the bivariate model, machine learning-based classifiers separated ASD from TD with a sensitivity of 73.8% (95% confidence interval (CI) 71.8–75.8%), a specificity of 74.8% (95% CI 72.3–77.1%), and area under the curve (AUC)/partial AUC (pAUC) of 0.803/0.765. A Summary Receiver Operating Characteristic (SROC) curve of the included studies—along with the estimated summary point, confidence region, and prediction region—is presented in Fig. 5a. Of the 132 samples, 40 were outside the 95% predictive region of the SROC curve, indicating heterogeneity. All the results obtained with the main analysis can be found in Supplementary Table S5.

Regression with year of publication did not affect sensitivity ($p = 0.250$) or specificity ($p = 0.283$), even segregating per type of ML technique.

#### ML technique and sample size

Considering the type of ML technique, only SVM and ANN classification tools were used in 5 or more articles. When analyzing the SVM studies, we obtained a sensitivity of 76.3% (95% CI 73.2–79.2%), a specificity of 77.5% (95% CI 73.7–80.8%), and AUC/pAUC of 0.832/0.748. The ANN studies had a sensitivity of 68.4% (95% CI 65–71.5%), a specificity of 70.2% (95% CI 66.2–73.9%), and AUC/pAUC of 0.743/0.582. The SROC curves for the studies using SVM and ANN are presented in Fig. 5b. We performed a subgroup analysis and found a significant difference between the sensitivities ($p = 0.002$) and the specificities ($p = 0.008$). However, after correction for multiple comparisons, only the difference in sensitivities remained significant.

We also analyzed the subtype of ML technique. For SVM, only L-SVM was used in five or more articles - sensitivity of 73.9% (95% CI 70.2-77.2%), specificity of 77.5% (95% CI 73.3–81.2%), and AUC/pAUC of 0.813/0.708—whereas for ANN the same happened with CNN - sensitivity of 66.7% (95% CI 63.3–69.9%), specificity of 70.1% (95% CI 66.3–73.7%), and AUC/pAUC of 0.732/0.565. Thus, we compared L-SVM with other types of SVM and CNN with other types of ANN. The comparison showed no effect on sensitivity or specificity (all $p>0.1$). Finally, the comparison with L-SVM against CNN indicated higher sensitivity ($p = 0.009$) and specificity ($p = 0.024$) in L-SVM studies. However, neither of those effects survived multiple comparisons correction.

**Figure 6:**
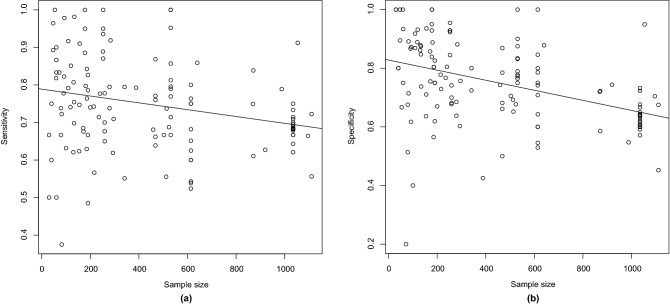
Linear regression models with sample size predicting sensitivity **(a)** and specificity **(b)** for all the studies. Created with R Statistics^125^ version 4.1.1 using the package mada^126^ version 0.5.10.

Regression with sample size as moderator showed a significant effect on both sensitivity ($p = 0.004$) and specificity ($p < 0.001$) when analyzing all the samples together, even considering multiple comparisons correction. Figure 6 shows the linear regression models with sample size predicting sensitivity and specificity and indicates that bigger sample sizes tend to obtain worse accuracies.

However, the same analysis segregating the studies per type of ML technique used indicated a significant effect on specificities ($p = 0.001$) and no impact on sensitivities ($p = 0.152$) for SVM studies, with worse specificities in studies with larger samples. Also, no significant effect was found for the ANN studies (all $p > 0.1$).

#### Subjects characteristics

No significant effects of sex (only males against males and females studies) or FIQ (neither considering the mean FIQ nor comparing studies that used only high-functioning subjects with the ones that used high- and low-functioning subjects) on sensitivity or specificity (all $p > 0.1$) were observed.

Regression with the mean age of the subjects did not affect sensitivity or specificity (all $p > 0.1$ considering ASD or TD groups). Comparison between samples with subjects under 18 years old and samples composed of individuals both under and above that age showed a significant difference between the specificities ($p = 0.020$) but no effect on sensitivity ($p = 0.225$), indicating higher specificity in studies that used only subjects under 18 y.o. (77.6—95% CI 73–81.6%—versus 70.5—95% CI 66.6–74.1%). This effect, however, did not survive multiple comparisons correction. Segregating per type of ML technique, only SVM had enough studies (17 studies and 37 samples) to conduct the analysis. Still, the results did not show any effect on sensitivity ($p = 0.790$) or specificity ($p = 0.427$). Sensitivity analysis considering other age thresholds (19, 20, 21) yielded the same conclusions (see Supplementary Table S6).

#### Sources of the samples

Subgroup analysis considering the database or source of the sample (ABIDE without version—comprising the studies that did not specify which version of ABIDE they were using—ABIDE I preprocessed or ABIDE I + ABIDE II) indicated a significant effect on the sensitivity when comparing ABIDE without version with ABIDE I preprocessed ($p = 0.046$) or ABIDE I + ABIDE II ($p = 0.043$). In both cases, the ABIDE without version group presented higher sensitivity (77.1%—95% CI 73.2–80.6%—versus 72%—95% CI 69–74.9%—and 69.2%—95% CI 65.8–72.4%—respectively). Nevertheless, this effect did not survive multiple comparisons. All the other analyses indicated no significant impact on sensitivity or specificity (all $p > 0.1$). The same analysis with SVM samples (ABIDE without version or ABIDE I preprocessed) did not indicate any effect on sensitivity ($p = 0.756$) or specificity ($p = 0.731$).

We conducted another analysis comparing the studies that used any version of ABIDE with studies that used databases or samples other than ABIDE (Own sample, NDAR, UMCD). The regression indicated higher sensitivity ($p = 0.024$) and specificity ($p = 0.045$) in studies that used databases or samples other than ABIDE (Sensitivity: 81.8%—95% CI 73.4–88.1%—versus 73.2%—95% CI 71.1–75.2%; Specificity: 83%—95% CI 72.5–90%—versus 74.1%—95% CI 71.6–76.5%), but the effects did not survive multiple comparisons.

#### Features definition

Subgroup analysis considering type of data (only rs-fMRI or rs-fMRI plus other data types) showed a significant difference between the sensitivities ($p = 0.002$) and the specificities ($p = 0.047$), indicating better results in studies that used other types of data together with rs-fMRI (Sensitivity: 84.7%—95% CI 78.5–89.4%— versus 72.8%—95% CI 70.6–74.8%; Specificity: 81%—95% CI 74.1–86.3%—versus 73.9%—95% CI 71.3–76.4%). However, after correction for multiple comparisons, only the difference on sensitivities remained significant.

**Figure 7:**
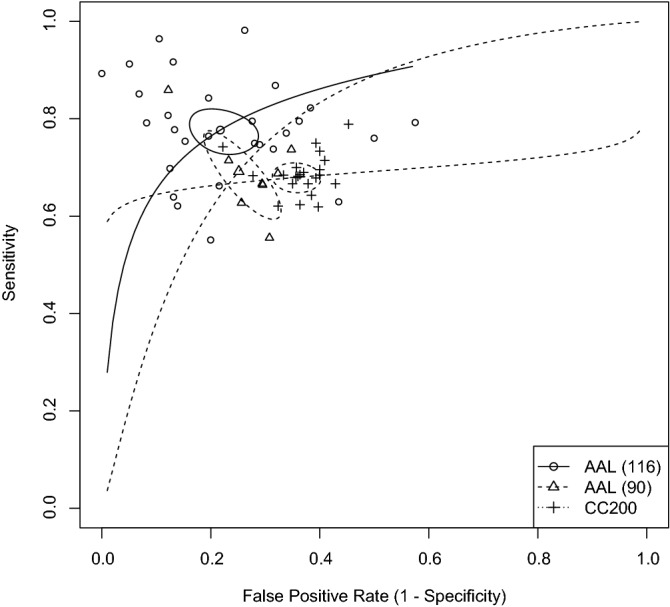
SROC curves of the studies using AAL90, AAL116, or CC200 with their summary estimates and confidence regions. Created with R Statistics^125^ version 4.1.1 using the package mada^126^ version 0.5.10.

Subgroup analysis considering the atlas used (AAL90, AAL116 or CC200), indicated that: studies using AAL90 obtained better specificity (74.9%—95% CI 68.7–80.1%; $p = 0.001$) than studies using CC200 (64.4%—95% CI 60.7–67.9%) but no significant effect was observed on the sensitivity ($p = 0.56$); studies using AAL116 obtained better sensitivity (77.7%—95% CI 73.7–81.2%; $p = 0.001$) and specificity (78.2%—95% CI 72.8–82.9%; $p < 0.001$) than studies using CC200 (Sensitivity: 68%—95% CI 65.4–70.4%); there was no significant effect on sensitivity ($p = 0.054$) or specificity ($p = 0.397$) between studies using AAL 116 or 90. Figure 7 shows the SROC curves for the studies using AAL90, AAL116 or CC200. All those effects remained significant after multiple comparisons correction.

Regression considering the number of ROIs used showed significant effects on sensitivity ($p = 0.043$) and specificity ($p = 0.018$). When segregating per ML technique, there was a significant effect on sensitivity ($p = 0.029$) for the SVM studies but no effect on specificity ($p = 0.089$) whereas for the ANN studies there was a significant effect on specificity ($p = 0.016$) but no effect on sensitivity ($p = 0.557$). For all the significant effects, the linear regression models indicated lower values of sensitivity/specificity as the number of regions increased. None of these effects survived multiple comparisons correction.

Subgroup analysis using the type of feature as moderator (PC, PC Fisher-transformed or others), indicated that: studies using PC (Fisher-transformed) obtained better sensitivity (76.7%—95% CI 71.3–81.3%; $p = 0.001$) and specificity (81%—95% CI 75.6–85.4%; $p < 0.001$) than studies using PC (Sensitivity: 68.9%—95% CI 66.8–70.9%; Specificity: 68.3%—95% CI 64.3–72.1%); similarly, studies using other features obtained better sensitivity (73.5%—95% CI 70.6–76.2%; $p = 0.031$) and specificity (74.7%—95% CI 71–78%; $p = 0.024$) than studies using PC; there was no significant effect on sensitivity ($p = 0.173$) or specificity ($p = 0.072$) between studies using PC (Fisher-transformed) and other features. Only the effects of the first analysis survived multiple comparisons.

#### QUADAS-2 analyses

Subgroup analysis considering the number of domains with low RoB in QUADAS-2 results (one or two) showed a significant difference between the specificities ($p < 0.001$) but no effect on sensitivity ($p = 0.236$), indicating higher specificity in studies that had only one domain with a low risk of bias (78.4%—95% CI 75.5–81.1%—versus 69.6%—95% CI 65.9–73%).

Analysis considering the RoB of the index test obtained from QUADAS-2 (high, unclear or low) indicated that: studies with unclear RoB obtained better sensitivity (80.5%—95% CI 76.1–84.2%; $p = 0.001$) than studies with high RoB (70.1%—95% CI 66.3–73.5%) but no significant effect was observed on the specificity ($p = 0.127$); studies with high RoB obtained better specificity (76.6%— 95% CI 73.2–79.8%; $p = 0.011$—did not survive multiple comparisons) than studies with low RoB (69.9%—95% CI 66.2–73.3%) but no significant effect was observed on the sensitivity ($p = 0.373$); studies with unclear RoB obtained better sensitivity ($p = 0.003$—did not survive multiple comparisons) and specificity (81.3%—95% CI 76.9–85%; $p < 0.001$) than studies with low RoB (Sensitivity: 72.1%—95% CI 69.6–74.4%).

We conducted another analysis splitting the studies with low RoB between the ones that performed a temporal (using data from newly recruited subjects) or geographic (using data collected by independent investigators at a different site) validation (5 articles and 35 samples) and the ones that performed a split-sample validation^127^. The conclusions were the same, except for the comparison between the studies with high RoB and the ones with low RoB (split-sample) that did not indicate a significant effect on sensitivity ($p = 0.291$) or specificity ($p = 0.148$). Also, there was no effect on sensitivity ($p = 0.451$) or specificity ($p = 0.441$) when comparing the studies that used a split-sample validation and the ones that used a temporal or geographic validation.

#### Sensitivity analyses

Sensitivity analysis including three more articles^5,68,69^—four new samples—that were initially excluded from the meta-analysis (as cited in “Studies included in the meta-analysis” section) indicated no significant change in overall sensitivity (73.7%—95% CI 71.7–75.6%) or specificity (75%— 95% CI 72.7–77.2%). All the results obtained with this sensitivity analysis can be found in Supplementary Table S7.

We repeated all the tests that the addition of those articles could impact. In general, their influence on the outcome was minor, but the conclusions from the meta-regression differed in three analyses. The regression with type of data as moderator showed the same conclusion for the sensitivities ($p = 0.002$) but no significant effect between the specificities ($p = 0.057$). The regression considering the database or source of the sample indicated no significant effect on sensitivity when comparing ABIDE with ABIDE I preprocessed ($p = 0.097$) or ABIDE I + ABIDE II ($p = 0.087$). Regression with atlas as moderator showed a significant effect on sensitivity ($p = 0.045$) between studies using AAL 116 or 90, indicating higher sensitivity in studies that used the AAL 116 (78%—95% CI 74.1–81.4%—versus 69.2%—95% CI 61.4-76%).

## Discussion

### ML techniques and sample size

As shown in Fig. 2, the number of publications in the area has been increasing following an exponential trend, and SVM was the most used classification technique, especially until 2018. Also, from the 54 samples that used SVM for classification, 33 of them used an L-SVM. The inherent characteristics of SVMs help them generalize well and deal with noisy, correlated features and high-dimensional data sets^128,129^.

However, we can observe an increasing number of studies using ANN techniques, making it the second most used technique in absolute numbers and the first when considering only articles published in 2019. Many ANN classifiers used deep-learning methods, especially the Convolutional Neural Network, corresponding to more than 35% of the ANN techniques.

Despite that, analysis considering the type of classification algorithm used indicated better results for SVM against ANN, especially on sensitivity. Also, the analysis comparing L-SVM and CNN resulted in the same conclusions (although not surviving multiple comparisons). In all those cases, the difference was about seven percentage points.

More complex models—such as deep-learning ones—tend to be more powerful but generally have more hyperparameters. Therefore, they are potentially more capable of explaining noise in the data and overfitting^130^. Also, it is not clear that those complex models always provide a significant advantage in practical performance. However, this can reflect the small number of samples available instead of indicating an absence of complicated relationships between features^131^.

We conducted a regression analysis to investigate the effect of the sample size on the results. Considering all the samples selected for the meta-analysis, we found worse results on both sensitivity and specificity by increasing the sample size. This same trend was also observed on specificities when considering only the SVM studies.

However, using only the ANN studies, we could not find any significant effect. Since a great part of the ANN methods were also deep-learning methods and more complex models may demand larger samples to avoid overfitting, our analysis suggests that ANN techniques may have an advantage when dealing with larger samples.

### Subjects characteristics

Several studies indicate gender, age, and IQ differences in autistic symptoms and impairments. For example, boys with ASD showed more restricted and repetitive behaviors than girls with ASD^132,133^; significant though modest effects of IQ and age indicated increased Autism severity with decreasing IQ and age^134^; also, lower socio-communicative symptoms were found in older compared to younger individuals^132^.

It is well known that ASD shows an imbalanced male–female ratio, and recent studies suggest values between 2:1 and 5:1^135–137^. There is also evidence that this ratio is lower in individuals with lower IQ^135,138^. Since most autism studies tend to follow this ratio or include only male participants, the underrepresentation of females may have led to an understanding of the disorder biased toward males^139^. Females are generally diagnosed later, and even with similar levels of severity of autistic traits, males are more likely to receive a diagnosis^140–142^.

Still, early detection and treatment would enormously benefit the individuals within the spectrum^13,14^. Therefore, it is essential to understand how those variables may affect classification accuracy to obtain a clinically useful ML diagnostic tool. In our analysis, we could not find any significant effect of the sex of the subjects or their FIQ on sensitivities or specificities. However, we must highlight some issues.

The regression considering the sex of the subjects compared the articles that used only male subjects with the ones without a sex restriction—whose samples were composed of males and females. Also, there were ten times fewer samples in the former compared to the latter subgroup. From the articles selected in the systematic review, only two^42,50^ performed tests considering different categories of gender. They obtained higher classification accuracies for females than males, even though the number of training samples for females was significantly lower.

Regarding the FIQ, we performed tests considering the mean FIQ and compared the samples composed of high-functioning subjects against those with both high- and low-functioning subjects. However, only 20% and 40% of the studies were included on those tests, respectively.

Regression with the mean age of the subjects did not affect sensitivity or specificity, but less than half of the articles presented enough information to be included in this analysis. On the other hand, analysis considering an adulthood threshold indicated higher specificity in studies that used only subjects under 18 y.o. when compared to studies without this restriction. This result is in accordance with some of the selected studies^42,65,66^, in which the adulthood segregation of the sample improved classification performance. However, the effect observed did not survive multiple comparisons, and the same analysis using only the SVM studies showed no significant effects (although it included almost three times fewer samples).

Analyzing the ABIDE—the most used database—we found a low proportion of child subjects: the ABIDE I do not include individuals below 7 years old whereas ABIDE II do not include individuals below 5 years old; in addition, from an analysis of the subjects that were available in both databases, less than 20% were below 10 years old (about 100 subjects in each one). The lack of younger individuals in those studies raises some questions. Those classifiers may be, in fact, detecting the consequences in terms of brain circuitry alterations of living with ASD instead of identifying the true roots of the disorder^30,143^.

As we can see, there is a lack of information regarding the characteristics of the subjects and samples included in many of the studies. Aggravating the problem, variables such as IQ, symptom severity, and handedness are missing for some sites of ABIDE^70^.

### Sources of the samples

As a heterogeneous and complex disorder, any ASD cohort is likely composed of ill-understood subtypes with different brain features. The use of large samples, such as provided by the ABIDE, can be helpful to address those issues^70^. Studies based on smaller datasets from a single site are composed of more homogeneous participants, reducing the generalizability of those models. Therefore, large multi-site datasets are needed to include a greater diversity of participants and obtain more reliable, robust diagnostic systems that generalize better to new data, revealing common features that contribute to classification^109,119,144,145^.

On the other hand, the massive use of a single database, as we saw in this study with ABIDE, end up limiting the interpretation and generalization of the analyses and results obtained.

We conducted analyses comparing the different versions of ABIDE used throughout the studies. At first, we found a significant effect on sensitivities, but this effect was not present in the sensitivity analysis or considering only the SVM studies. It is noteworthy that the ABIDE without version group is composed of samples from the ABIDE I, ABIDE I preprocessed, or ABIDE II, but the studies did not specify which version they were using.

When comparing the studies that used any version of ABIDE (121 samples) with studies that used databases or samples other than ABIDE (9 samples), the analysis indicated higher sensitivity and specificity in the studies of the first subgroup. This may reflect the greater size and diversity of the ABIDE compared to the other sources. However, the effect did not survive multiple comparisons, and we highlight the imbalance between those subgroups.

It is also vital to notice that, while most individuals with ASD live in low- and middle-income countries^10,12^, all of the samples used in this meta-analysis were from high-income countries, and only one of them was not from North America or Europe.

Therefore, it is of utmost importance that more diverse datasets and studies be created and conducted. The applicability of the results obtained so far needs to be tested and confirmed across different cultures and social classes. Also, larger and more diverse samples would allow studies using restricted samples (such as low-motion data or samples composed entirely of female subjects) to obtain more reliable and robust results by selecting a bigger number of participants.

### Features definition

Even though the focus of our research was the ML diagnostic tools that used rs-fMRI for classification, some of the selected studies used other types of data together with rs-fMRI aiming to obtain better results by complementing the information available. In general, we found two types of complementary data: phenotypic information such as age and sex; other brain imaging data, specially sMRI. As there were not enough articles in each of these subgroups, we compared the studies that used only rs-fMRI data with those that used any other type of data together with rs-fMRI. Our results indicated a higher specificity in the latter case.

Different brain images provide different views of the same brain and may reveal hidden evidence of ASD that is not available by using a single imaging modality^121^. However, we must highlight that investigation of the effect of combining different types of data in the classification is not the main objective of this study.

Most of the studies defined ROIs using a priori atlases. However, those atlases are often selected arbitrarily in the rs-fMRI community^33^. Therefore, we conducted a subgroup analysis with the atlas used as moderator.

Both versions of the AAL obtained significantly better results than the CC200, but there was no significant difference between them. The sensitivity analysis, however, indicated better sensitivity for studies using the AAL116. We must also highlight that, in both cases, the p-values of the comparison between the versions of the AAL were close to the threshold of 0.05, and those results should be taken with extra caution.

We made a regression analysis considering the number of ROIs used throughout the studies. Our results indicated smaller accuracies as the number of regions used increased—more specifically, worse sensitivities for the SVM studies and worse specificities for the ANN studies. This may be because more ROIs generally result in more features available. Using a large number of features relative to the number of data samples can cause classifiers to overfit^19^. However, the effect did not survive multiple comparisons.

The variety of choices in data processing adds to the variability of the results obtained in the studies of the field^22,146^. Therefore, we conducted a subgroup analysis considering the type of feature used. Our results indicated a significant advantage on sensitivity and specificity to the studies using the Fisher-transformed version of the PC against the studies using it without modifications. The studies using other features showed the same benefit against the PC ones (although not surviving multiple comparisons). Finally, even though the studies using the PC Fisher-transformed obtained better summary estimates for sensitivity and specificity, their comparison against the studies using other features did not indicate any significant effect—which is not a surprise considering the great variety in the latter group.

### QUADAS-2 analyses

Bias and variation are often present in diagnostic test accuracy studies. Therefore, they need to be detected and assessed to understand the validity of the meta-analytic results obtained^147,148^. Through QUADAS-2 application (see Fig. 4), we found many studies with high RoB on the patient selection domain, basically due to the selection of subjects in restricted intervals of age and IQ or the exclusion of female subjects. The effect of those variables on the classification results was already discussed in “Subjects characteristics” section.

Beyond that, we can see that many studies were assessed to have an unclear RoB, especially on the patient selection and flow and timing domains. This reinforces the necessity to present more detailed information regarding the characteristics of the subjects and samples included in the publications.

We conducted some analysis using the QUADAS-2 results. The first one considered the number of domains with low RoB in each study and indicated higher specificity in studies with only one low RoB domain. We also performed a subgroup analysis using the index test domain results. As expected, studies with high or unclear RoB obtained significantly better results than studies with low RoB. We also found better outcomes for the studies with unclear RoB in comparison to those with high RoB. We suppose that a significant part of those studies assessed as unclear should have been assessed as high, but there was not enough information to conclude that. This also indicates a bias of overestimation—at least for the specificities—on the studies that do not apply their best models to independent sets after testing different numbers of features or atlases.

For the last analysis, we separated the low RoB category into the articles that performed a temporal or geographic validation and those using a split-sample validation^127^. Even though the latter obtained better summary estimates than the former, there was no significant effect between their sensitivities or specificities.

From the clinical standpoint, complete external validation (temporal or geographic) is preferred. Split-sample validation would not accurately assess the generalizability of a model. In contrast, geographic validation is helpful for this purpose since it may be performed with different technical parameters at different sites^127^. Our analysis did not indicate a significant difference between the results using each of these types of validation. However, we must highlight that only 5 articles with low RoB by the index test domain performed a complete external validation, and it is still the more reliable approach to assess generalizability.

### Clinical validity

At present, ASD diagnosis is based on behavioral criteria, being vulnerable to subjectivity and interpretative bias. Also, less experienced clinicians seem to have more problems with the challenges of this complex diagnostic process^17,149^.

The diagnostic utility and discriminative ability of the ADOS-G and the ADI-R were assessed using a clinical population of children^150^. The results indicated approximately 75% of agreement with the qualified multidisciplinary team diagnoses, and most inconsistencies were false positives. The accuracy and validity of the ADOS-2 and ADI-R in diagnosing ASD in adults without an intellectual disability were also evaluated^149^. The original algorithm of ADOS-2 Module 4 obtained 85.9% and 82.9%, whereas its revised algorithm obtained 87.2% and 74.3% of sensitivity and specificity, respectively. On the other hand, the ADI-R got 43.1% of sensitivity and 94.7% of specificity.

Considering all the studies selected for the meta-analysis, we found summary sensitivity and specificity of 73.8% and 74.8%, respectively, for the ASD diagnosis using rs-fMRI and ML classifiers. Also, the AUC/pAUC of 0.803/0.765 indicates values between acceptable and excellent^151^ (0.5: no discrimination; 0.7–0.79: acceptable; 0.8–0.89: excellent; $\ge$ 0.9 outstanding). If we look at the analysis considering only the SVM studies, those results were even better, with sensitivity and specificity above 76% and AUC of 0.832. Also, we found acceptable AUC values within the articles that presented lower RoB and, therefore, more reliable results.

Even though these results seem promising and somewhat close to the ones obtained with diagnostic tools currently used for ASD, there is a long journey ahead before those ML algorithms could be used in clinical practice. First of all, the limitation found in the literature regarding the almost exclusive use of ABIDE as a source of samples also limit the interpretability of the quantitative results in this meta-analysis.

Beyond that, the articles included in our analysis presented a great variety of features extracted and selected, classifiers used, and validation approaches applied. Thus, the summary estimates that we obtained show the overall potential of those procedures but do not indicate a specific one to be used in clinical practice. It would even be possible to use different classifiers for subjects with different characteristics—such as sex and age—similarly to what happens with the modules of ADOS-2.

Taking the variety of neurodevelopmental etiologies believed to exist within the ASD population, there may not be an exceptional biomarker to diagnose the disorder^4,5^. Perhaps the classifiers must consider different biomarkers for different etiologies, partitioning the ASD into more than a single class^152^.

The decision on ASD diagnosis concerns the relationship established by the individual with their environment, which can lead to significant barriers to their quality of life and not to a specific condition in itself^153^. Therefore, a binary classifier of ASD vs. non-ASD might not be clinically helpful by not considering the environmental factors or other similarly presenting conditions. Still, the margin of doubt regarding the impairment on quality of life is limited to milder ranges of the disorder, reducing the probability that the individual does not meet the diagnostic criteria as the autistic traits accumulate. Suppose these classifiers reliably demonstrate their consistency with formal clinical diagnostic. In that case, we could take advantage of these more efficient and possibly more disseminated and democratic tools to build a scenario of access to diagnosis to all those who need the resulting social support.

Another issue to consider is the difficulty of many children and low-functioning individuals with ASD to tolerate fMRI scans, which may explain their underrepresentation within the samples analyzed. However, the application of fMRI during natural sleep can help to overcome this limitation^154,155^. The only article in this review that used subjects below five years of age^89^ applied this methodology to obtain data from 6-month-old infants, with classification results above 80%.

Many questions need to be assessed to define the clinical validity of those procedures. It includes the underrepresentation of females in research and clinical practice, the effects of subject’s IQ and age, the lack of such information in many studies, and the necessity of larger and more diverse samples to confirm the generalizability of the classification tools.

### Limitations

Some limitations must be considered. The biggest one is the sample overlap between the studies, especially considering the lack of information on the patient selection process and the large number of studies that used the ABIDE database. Sample overlap induces a correlation structure among empirical outcomes, which, if not accounted for, can harm the statistical properties of meta-analysis methods and result in higher rates of false positives^156^. Thus, it is not clear to which extent this overlap could bias the results obtained. Furthermore, due to the tremendous heterogeneity of ASD, this high degree of overlap may limit the interpretability and generalizability of our analysis.

Despite that, we highlight that all the significant results obtained in our analyses were reasonable and in line with the literature, as we discussed in the previous subsections. Also, we clearly stated this limitation throughout the study and hope that it serves as a guide to future works, eventually reaching a state where more robust analyses can be done.

Considering the significant heterogeneity within the selected publications, the summary estimates obtained through the meta-analysis have to be interpreted with caution and in light of the methodologic quality of the studies, as previously discussed. Most studies provided only limited information regarding the patients samples and their clinical characteristics. However, detailed information about the participants’ disease status, symptoms, current medication, history of interventions, or comorbidities is crucial for evaluating the potential of the proposed models to be applied in clinical practice^70,157^. Thus, the impact of those variables on classification accuracy needs to be better investigated.

The studies included in our analysis identified ASD-distinctive brain patterns as compared to healthy volunteers. Nevertheless, it is critical to investigate the patterns of brain abnormalities that differentiate between different psychiatric disorders. Also, the results obtained in this meta-analysis do not apply to individuals below five years of age since almost none of the studies included individuals with such low age.

In addition, some methodological steps were not investigated in our analyses, such as the data preprocessing and feature selection procedures. Those aspects still need to be assessed to define their effects on classification accuracy.

### Recommendations

Based on our results, we recommend that future studies obtain their features using the PC Fisher-transformed instead of using it without modifications. Also, the AAL116 seems to be a good choice of atlas, and we encourage studies to explore other types of data to complement rs-fMRI.

In the face of large samples, ANN techniques seem to have an advantage compared to SVM. However, considering the limitations of our study and the other methods not analyzed, we think it is a bit premature to recommend any of these techniques. For example, two articles^70,92^ using RF obtained results around 90%, and it would be interesting to include this technique in future analyses.

It would be of great value for future publications in this field to apply the following best practices, when possible: report, at least, measures of sensitivity and specificity; present detailed information of the subjects and samples used (sex, age, IQ, number of TD and ASD subjects, etc.); inform the reference standard used and how the patients were selected, even if the sample came from an existing database; after conducting all the tests, apply the best model to an independent sample, preferably using a temporal or geographic validation; use more diverse samples, especially from low- and middle-income countries; if the samples came from an existing database, specify which subjects were included.

### Conclusions

We performed a comprehensive analysis on the literature of ML classifiers using rs-fMRI data for ASD diagnosis, indicating promising pathways and questions to be addressed. Our results showed overall sensitivity and specificity estimates of around 75%. We found better accuracy for SVM classifiers, but ANN techniques may have an advantage in dealing with larger samples. Also, the use of other types of data to complement rs-fMRI information seems to be promising.

To the best of our knowledge, this is the first meta-analysis focused on the topic, and we reiterate the availability of all extracted data. However, given the many limitations indicated in our study and the poor methodological quality found in a great part of the selected articles, further well-designed studies are warranted to extend the potential use of those classification algorithms to clinical settings, and the quantitative meta-analytical results presented here should be taken with caution.

## Methods

### Search strategy

The articles used in this review were found through four digital libraries: Scopus, El Compendex, PubMed—NCBI, and IEEE Xplore. Considering that the El Compendex and PubMed NCBI libraries resulted in many duplicated articles (approximately 73% and 91% of the articles, respectively), we decided not to include other libraries in the search and find out more studies through the snowballing.

The search expression was iteratively defined using keywords considered appropriate. We analyzed the titles and abstracts of the publications found through the searches to determine whether they were related or not to the purpose of this study. Based on that, we refined the search expression and obtained the final version presented below:

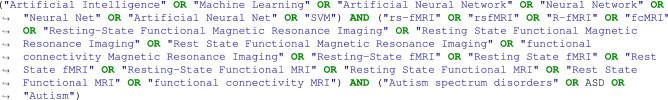



We started using other expressions related to ASD as defined by the Diagnostic and Statistical Manual of mental disorders 4th edition (DSM-IV)^158^ to possibly include articles published before 2013—when the DSM-V^3^ was first published. These expressions were: “Pervasive Development Disorders”; “PDD”; “Autistic Disorder”; “Asperger’s Disorder”; “Asperger”; “Childhood Disintegrative Disorder”; “PDD-NOS”. However, the addition of these terms only resulted in two new articles that were not related to the purpose of this study. Therefore, we decided to simplify the expression by removing those terms.

The search was carried out in two parts. First, we searched for articles published between January 1, 2010, and December 7, 2018, the date of the last search conducted. The string was applied directly in the digital libraries El Compendex and PubMed. The advanced mode was used for Scopus, and the search was specified for title, abstract, and keywords. Likewise, the advanced mode was used for IEEE Xplore, but the search was specified for full text. The start date was defined considering that, during the tests with the string, only one article published before 2010 was found, and it did not fulfill the criteria to be included in this study. Furthermore, the use of the snowballing technique should retrieve the most relevant papers published before this date.

After the first search, the development of the study took longer than expected. Therefore, a second search was performed to keep the study updated. We searched for articles published between December 7, 2018, and April 3, 2020, the last search date. The string was applied to the digital libraries using the same process as in the first search. The only exception was the IEEE Xplore, for which we used the command search instead of the advanced mode, and the search was specified for full text and metadata.

### Study selection

First, a triage process was applied to the non-duplicate publications. Three authors (C.P.S., E.A.C., I.D.R.) submitted each paper to a selection based on specific inclusion and exclusion criteria previously defined (see Supplementary Table S8). However, some exclusion criteria needed to be created or adjusted during the selection for better classification.

Generally speaking, we included publications that used ML techniques to classify subjects between ASD and TD based only on rs-fMRI or based on rs-fMRI together with other types of data. Guidelines for applying ML techniques in the classification of brain images were included if they presented classification results regarding rs-fMRI and ASD. Also, publications focused on distinguishing ASD from other disorders were included if they classified ASD vs. TD.

The criteria were applied based on the abstracts of the studies. When it was not sufficient, a superficial reading of the entire article was carried out—it is worth noting that this was conducted only for a pre-selection of the articles. The papers were selected if at least one of the researchers concluded it should be. Then, the same three researchers performed a new assessment to confirm the selection. In this step, each paper selected was read carefully to determine if it fulfilled three requirements: (1) used rs-fMRI data; (2) performed a classification between ASD and TD; (3) the classification was performed using an ML technique. If at least one of those requirements was not fulfilled, the article was excluded from the study.

### Data extraction

Three authors (C.P.S., E.A.C., I.D.R.) used a standardized data extraction sheet to collect data from all included studies (see Supplementary Table S4 online). We extracted the source and type of the data, sample size, if the study included both males and females, average age and FIQ of the subjects, preprocessing steps, feature extraction and selection procedures, the validation process, classifiers used, outcomes reported, main results (accuracy, sensitivity, specificity, and measures of TP, TN, FP, and FN), other tests performed, and important brain areas.

We extracted/calculated only one result from each independent sample in a study. Since the majority of the publications presented multiple results from different tests, the main results were selected according to the following criteria: results from the classification method proposed in the article were prioritized; results presenting enough information to conduct the meta-analysis (measures of TP, TN, FP, and FN, number of ASD and TD subjects in the test set) were prioritized; results using only rs-fMRI data were prioritized; results using a hold-out test set, an inter-site (leave-one-site-out) approach or a train/validation/test procedure were prioritized; tests using larger samples were prioritized; if the study presented results using different numbers of folds for the cross-validation, tenfold was prioritized (the most common approach); finally, the results with higher accuracy were prioritized.

### Snowballing

Snowballing means systematically searching for primary studies based on references to and from other studies. Since we limited our research to the date of the last search conducted, we only performed a backward snowballing^31^. The goal was to broaden the scope of this work and include the maximum number of related articles, especially those before 2010, if any.

As the selected articles were analyzed, we looked for references that could be included in this systematic review according to the inclusion/exclusion criteria. It resulted in many duplicated articles, so we decided not to re-apply the snowballing technique. Also, the new articles found went through the same selection process presented before.

### Quality assessment

One author (C.P.S.) assessed methodological quality using the QUADAS-2^32^—the currently recommended tool for a systematic review of diagnostic accuracy studies^147,159^.

QUADAS-2 assesses study quality in four key domains: patient selection, index test, reference standard, and flow and timing. All the domains are assessed in terms of RoB, and the first three are also evaluated in terms of concerns about applicability (the concern that a study does not match the review question)^32^.

The tool was tailored by two authors (C.P.S., E.A.C.). After defining the signaling questions and review-specific guidance, both authors applied the tool using five articles. The answers to the signaling questions and the risks of bias/applicability were compared, and any disagreement was discussed to reach a consensus. We maintained the core signaling questions for each domain as defined by the QUADAS-2^32^ except for the index test domain, for which we defined the level of RoB by reviewing the validation process used to obtain the classification accuracy.

Studies using a nested cross-validation procedure or a hold-out set for testing the proposed classification algorithms were assessed as having low RoB. If a study presented their results per number of features, per atlas used or similar, but the best model was not applied to an independent set, there was a high RoB. Studies using a cross-validation scheme without providing any further information were considered as having an unclear RoB. The applicability concerns of the same domain were based on the type of data used. Studies using other data types beyond rs-fMRI were assessed as having high concerns unless the data used would be available in a real application (e.g., age or gender).

The QUADAS-2 tool was applied two times. In the first, all articles were assessed as a whole. In the second, only the papers selected for the meta-analysis were evaluated, considering the main results (as defined in “Data extraction” section) used for the statistical analysis. In both cases, the information used to reach the judgment of each of the domains was recorded to make the rating transparent and facilitate discussion.

### Statistical analysis

Studies were eligible for inclusion in the quantitative meta-analysis if TP, TN, FP, and FN measures were available or if the data allowed for their calculation. Therefore, we excluded studies that: did not report sensitivity/specificity (nor equivalent metrics); did not present enough information regarding the number of TD and ASD subjects on the test set. We also chose not to include articles with results reported only through bar charts nor RF studies without enough information on their OOB results (see “General study characteristics” section for more details). However, those three articles were included in a sensitivity analysis. The TP/TN/FP/FN values were extracted or calculated from each independent sample in a study according to the criteria defined in “Data extraction” section.

To avoid bias, handling sample overlap between the studies is necessary, possibly excluding samples with considerable overlap. However, the majority of the studies selected in this review extracted their samples from the ABIDE database. Thereby, we have a lot of potential overlapping samples. At the same time, there is little information concerning the exact individuals used in each study to conclude the real extent of the overlap. Excluding all the potential overlapping samples would make it difficult to perform a meta-analysis since only a few results would remain. Furthermore, we can consider that the studies vary considerably regarding characteristics such as the preprocessing, features, and classification techniques used. Thus,this overlap could not be accounted for and we decided to use all the results regardless of it.

The statistical analysis was performed using the open-source package mada^126^ version 0.5.10 in R Statistics^125^ version 4.1.1. A coupled forest plot of sensitivity and specificity was created using RevMan version 5.3^160^. SROC curves, summary estimates of sensitivity and specificity, and the corresponding 95% CIs were calculated by the bivariate model of Reitsma et al.^161^. Prediction region, AUC, and pAUC were also obtained. Studies that were visually deviant from the 95% prediction region on the SROC curves were considered heterogeneous^162^.

Subgroup analysis and bivariate meta-regression with potential covariables were performed to reduce any heterogeneity noted between the studies. The ML technique used, year of publication, sample size, type of data, source of the sample, atlas used, number of ROIs, QUADAS-2 results, type of features, and sex, IQ, and age of the subjects were investigated. Knowing that the bivariate model has five parameters^162^, we considered $n = 5$ the minimum number of studies to justify a separate meta-analysis. All tests were based on a 2-sided significance level of $p = 0.05$.

Since we conducted many tests of significance, we applied the Bonferroni correction^163^ to account for multiple comparisons. We considered different corrections for different families of tests^164^. Therefore, for subgroup analysis (30 tests) the corrected significance level is $p = 0.002$ while for meta-regression (13 tests) the corrected significance level is $p = 0.004$.

In sensitivity analysis, three studies that were initially excluded from the meta-analysis were included to verify the robustness of the results. Also, we investigated the effect of the age of the subjects considering different adulthood thresholds (18–21 years old).

Publication bias was not assessed in our analysis, as there are currently no statistically adequate models in the field of meta-analysis of DTA studies, and further research is required^162^.

## Supplementary Information

Supplementary Figure S1.

Supplementary Table S1.

Supplementary Table S2.

Supplementary Table S3.

Supplementary Table S4.

Supplementary Table S5.

Supplementary Table S6.

Supplementary Table S7.

Supplementary Table S8.

## Data Availability

The datasets analyzed during the current study are available in the ABIDE I and ABIDE II repositories, https://fcon_1000.projects.nitrc.org/indi/abide/.
