# Supplementary material for: rs-fMRI and machine learning for ASD diagnosis: a systematic review and meta-analysis
Source: Sci Rep. 2022 Apr 11;12:6030. doi: 10.1038/s41598-022-09821-6 (PMC9001715; doi:10.1038/s41598-022-09821-6)

**Supplementary Figure S1.** Paired forest plot of all samples included in the meta-analysis. Created using RevMan version 5.3.


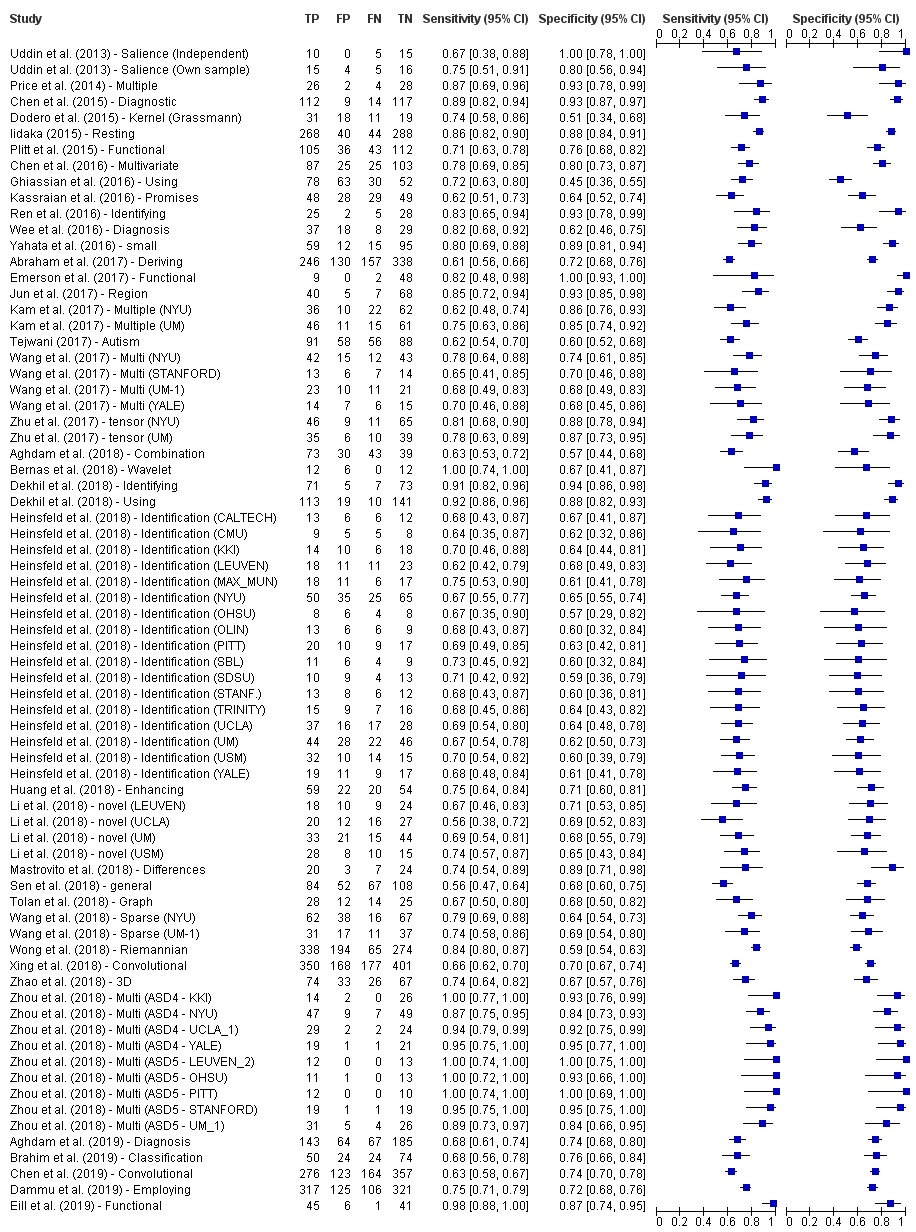


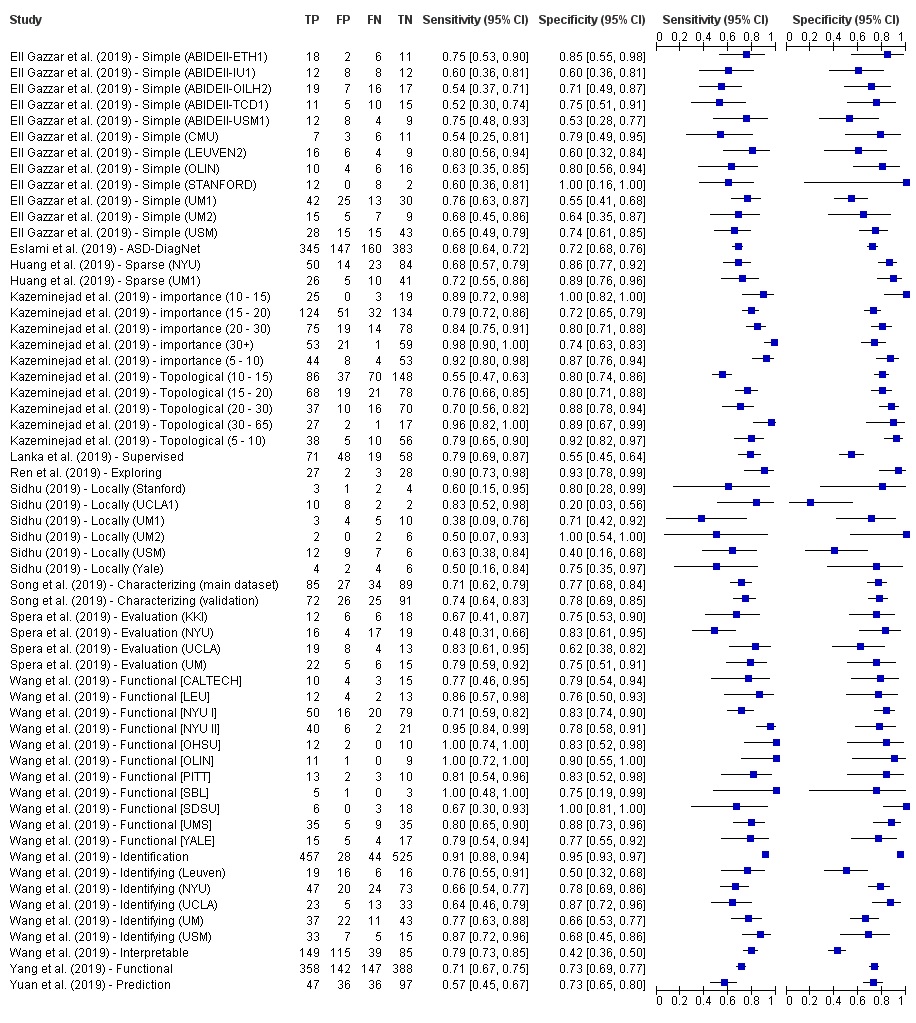

Supplement: Supplementary file 1 — Supplementary Figure S1. [file 41598_2022_9821_MOESM1_ESM.docx]
